# Evaluation of acinetobacter baumannii pneumonia among critically ill patients in a tertiary care hospital in Saudi Arabia

**DOI:** 10.1016/j.heliyon.2020.e03976

**Published:** 2020-05-20

**Authors:** Duaa Alsulaiman, Nada Al-Hamed, Anhar Alziadi, Alhanouf Almalaihi, Mustafa Alessa, Rania Khalil, Royes Joseph, Dhfer Alshayban

**Affiliations:** a College of Clinical Pharmacy, Imam Abdulrahman Bin Faisal University; b Alamal Complex for Mental Health; c Essex Partnership University NHS Foundation Trust

**Keywords:** Microbiology, Bacteria, Antimicrobial, Infectious disease, Medical microbiology, Pharmacology, Pneumonia, *Acinetobacter baumannii*, Colistin

## Abstract

Microbiology; Bacteria; Antimicrobial; Infectious disease; Medical microbiology; Pharmacology; Pneumonia; *Acinetobacter baumannii*; Colistin.

## 1 Introduction

Pneumonia is one of the types of lower respiratory tract infections (LRTI) causing mild to severe illness in people of all ages. Pneumonia is classified into different types including community acquired pneumonia (CAP), hospital acquired pneumonia (HAP), and ventilator associated pneumonia (VAP). Several types of pathogens cause nosocomial infections, *Acinetobacter baumannii* is one of the most significant nosocomial bacterial pathogens in hospital-acquired infections and especially in the setting of intensive care units (ICUs). This pathogen can cause many types of infections such as meningitis, bacteremia, skin infection, urinary tract infection and most commonly hospital acquired pneumonia [1, 2, 3, 4]. *A.bumannii* is an aerobic gram-negative bacilli that can develop resistance to several antibiotics, which results in limited treatment options leaving only the last resort antibiotics such as colistin-based, sulbactam-based and tigecycline-based therapy [4, 5]. A systematic review evaluated the rates of VAP in developing countries and showed that *A.baumannii* was the second most common pathogen associated with VAP [6]. Likewise, a national prospective surveillance study found similar results suggesting that *A.baumannii* is one of the main causative agents of VAP among critically ill patients [7].

The increase rates of pneumonia due to *Acinetobacter Bumanni* infection among critically ill patients can be attributed to multiple prognostic factors such as recipients of mechanical ventilation, prolonged length of hospitalization, exposure to the intensive care unit, surgical and postsurgical procedures, invasive procedures, comorbidities such as chronic obstructive pulmonary disease (COPD), cardiac diseases, advanced age and high Chronic Health Evaluation II (APACHE II) score. Most importantly, broad spectrum antibiotics use and a delay in the administration of appropriate antibiotic therapy increase the rate of infection as well [8, 9, 10].

Several studies emphasized that those patients with ventilator associated pneumonia (VAP) caused by *Acinetobacter baumannii* have a high risk of mortality. Some studies attributed this increased risk to antibiotics resistance, specifically carbapenem-resistance which led to increased rates of mortality from 20% to 50% along with prolonged hospitalization and ICU length of stay [11, 12, 13]. Additionally, treating such infections especially if there are high resistance rates is an economic burden to the respective institution [14].

We sought to conduct this retrospective study with the purpose of evaluating the antimicrobial therapy, length of hospital stay and mortality rate of pneumonia caused by *A.baumannii* among critically ill patients at a tertiary care hospital in Saudi Arabia.

## 2 Methods

This is a retrospective chart review study. It was conducted between January 2017 and December 2017. There was no formal sample size calculation carried out as this was a descriptive study that examined medical charts over one year. Generally, studies suggest 10 cases per variable is acceptable for obtaining clinically meaningful results. This study was done in an academic tertiary care hospital, King Fahad Hospital of the University (KFUH) in Saudi Arabia. The study population included all patients admitted to the ICU at KFHU during the study period. KFUH consists of 440 beds, 42 beds are located within surgical intensive care unit (SICU) and medical intensive care unit (MICU) which are the two adults ICUs in the hospital.

All ICU hospitalized patients admitted to either SICU or MICU and had *A. baumannii* infection during the year of 2017 were screened. The inclusion criteria were: 1) age >14 years old; 2) patients who were admitted to the SICU or MICU with a pneumonia infection caused by *Acinetobacter baumannii.* Exclusion criteria were: 1) patients on immunosuppressant agents; 2)immunocompromised patients such as patients with lymphoma, leukemia, AIDS, and thyroid cancer. Patients' information collected from the medical charts to include demographic characteristics, admission reasons and comorbidities. Defined risk factors, which were considered per investigators based on previous studies, included use catheters (central venous catheter or indwelling catheter (Foley Catheter)), dialysis (hemodialysis, peritoneal dialysis or continuous renal replacement therapy), mechanical ventilation, length of stay and use of antibiotics in the last 90's days. The highest SrCr and WBCs during the first 10 days of the infection were also collected and reported. The microbiology sample was identified. Sensitivity tests were obtained for available antibiotics for testing per our microbiology which includes: ceftizidime, cefepiem, ciprofloxacin, levofloxacin, meropenem, piperacillin/tazobactam, tigecycline, amikacin, ampicillin, gentamicin, and trimethoprim/sulfamethoxazole. Antibiotic therapy used in treatment of the *A. baumannii* pneumonia were also collected and identified as well as the outcomes of each patient. The ethical approval was obtained from the Deanship of Scientific Research, Institutional Review Board (IRB). Data were summarized using descriptive statistics. Percentages and frequencies were used for the categorical variables, while median was calculated for the continuous variables. Data management and analyses were carried out using SPSS Statistics 24.0 (IBM Corp, Armonk, NY).

## 3 Results

**Figure 1:**
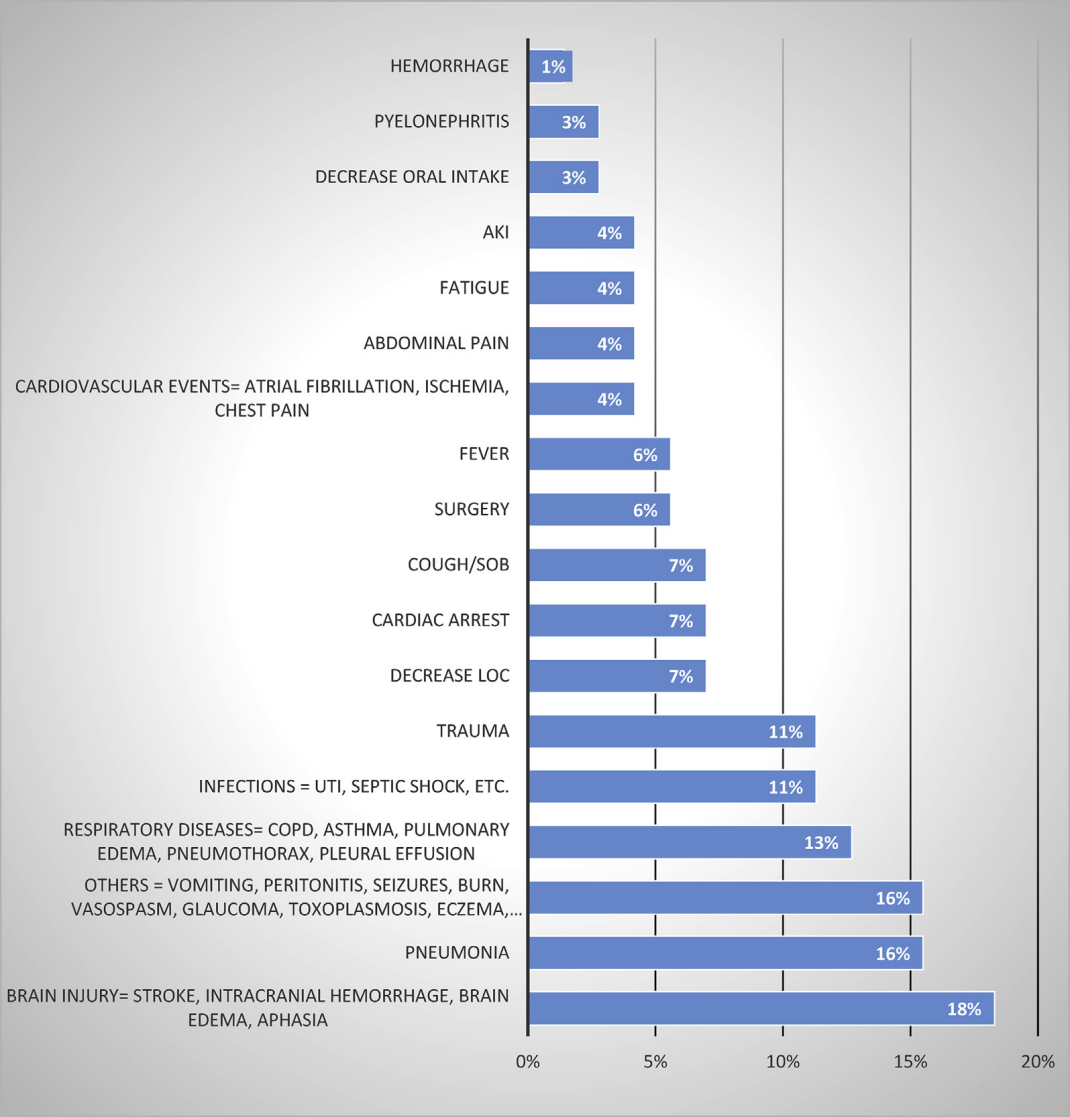
Hospital Admission Reasons. AKI, Acute Kidney Injury; SOB, Shortness of Breath; LOC, Level of consciousness; UTI, Urinary Tract Infection; COPD, Chronic Obstructive Pulmonary Disease.

**Figure 2:**
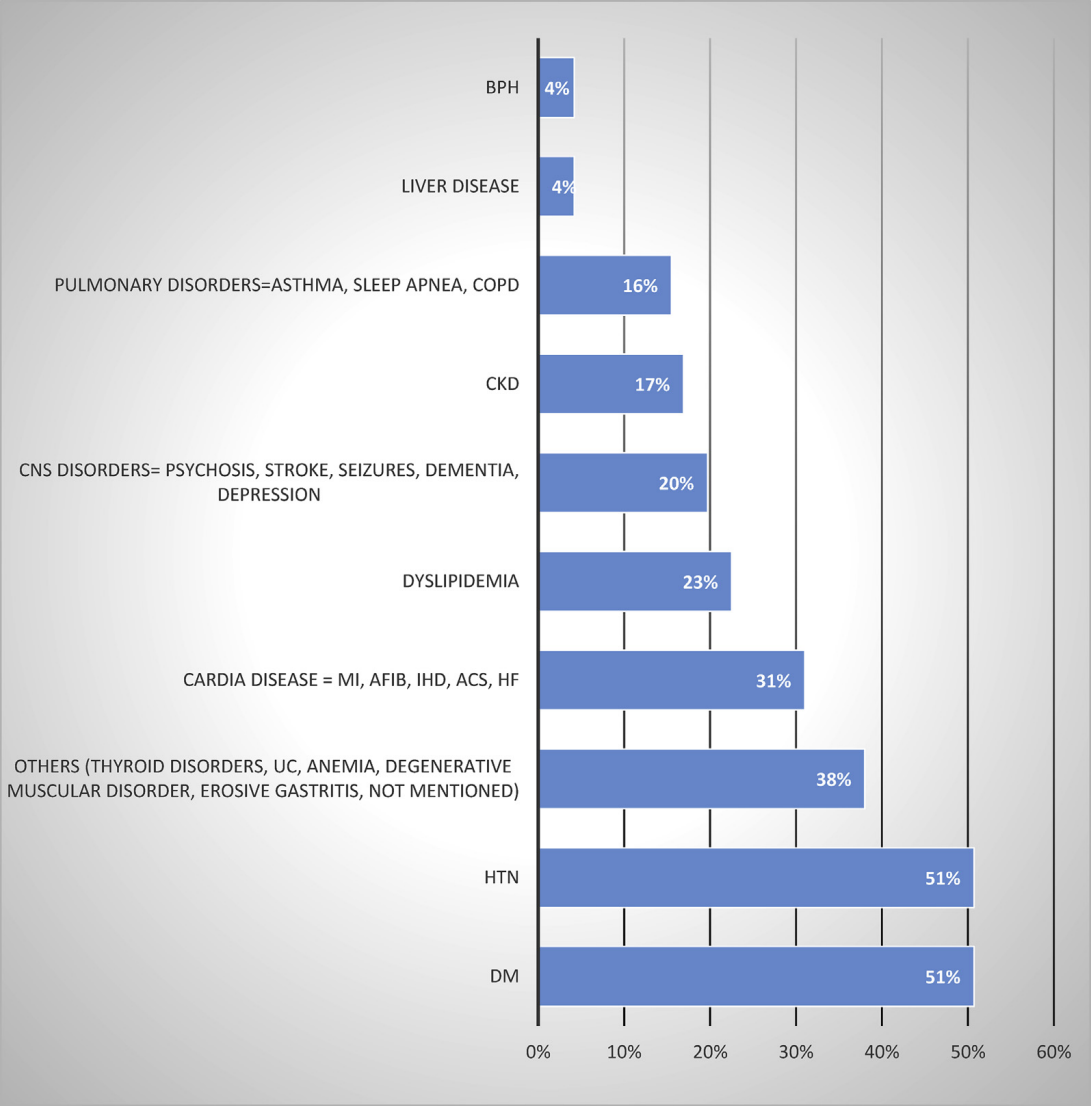
Comorbidities conditions. BPH, Benign Prostatic Hyperplasia; CKD, Chronic Kidney Disease; MI, Myocardial Infarction; A Fib, Atrial Fibrillation; IHD, Ischemic Heart Disease; ACS, Acute Coronary Syndrome”Angina”; HF, Heart Failure; UC, Ulcerative Colitis; HTN, hypertension; DM, Diabetes Mellitus.

This retrospective study consisted of 71 patients, 44 (62%) were male patients. The median age was 65 years. Prior to starting treatment, the median white blood cells was 14.9 × 109/L, the median SrCr was 1.57 mg/dl (Table 1). The most reported admission reasons were brain injury events (stroke, intracranial hemorrhage) and pneumonia (Figure 1), and the most reported comorbidities were diabetes mellitus and hypertension (Figure 2).

**Table 1:** Baseline characteristics.

Male n (%)	44 (62%)
	Median
Age (years)	65.5
WBC (/L)	14.90 × 10^9^
SrCr (mg/dl)	1.57

The common defined risk factors which occurred prior to the date of infection among the patients were previous use of antibiotics within the past 90 days (84%), use of mechanical ventilation (73%), use of any catheter (63%), and being on dialysis (17%) (Table 2).

**Table 2:** Risk factors.

Risk factors	*n*	(%)
Previous use of Antibiotics in the last 90 days	60	84%
Mechanical Ventilation	52	73%
Use of catheter [1]	45	63%
Dialysis [2]	12	17%

**Figure 3:**
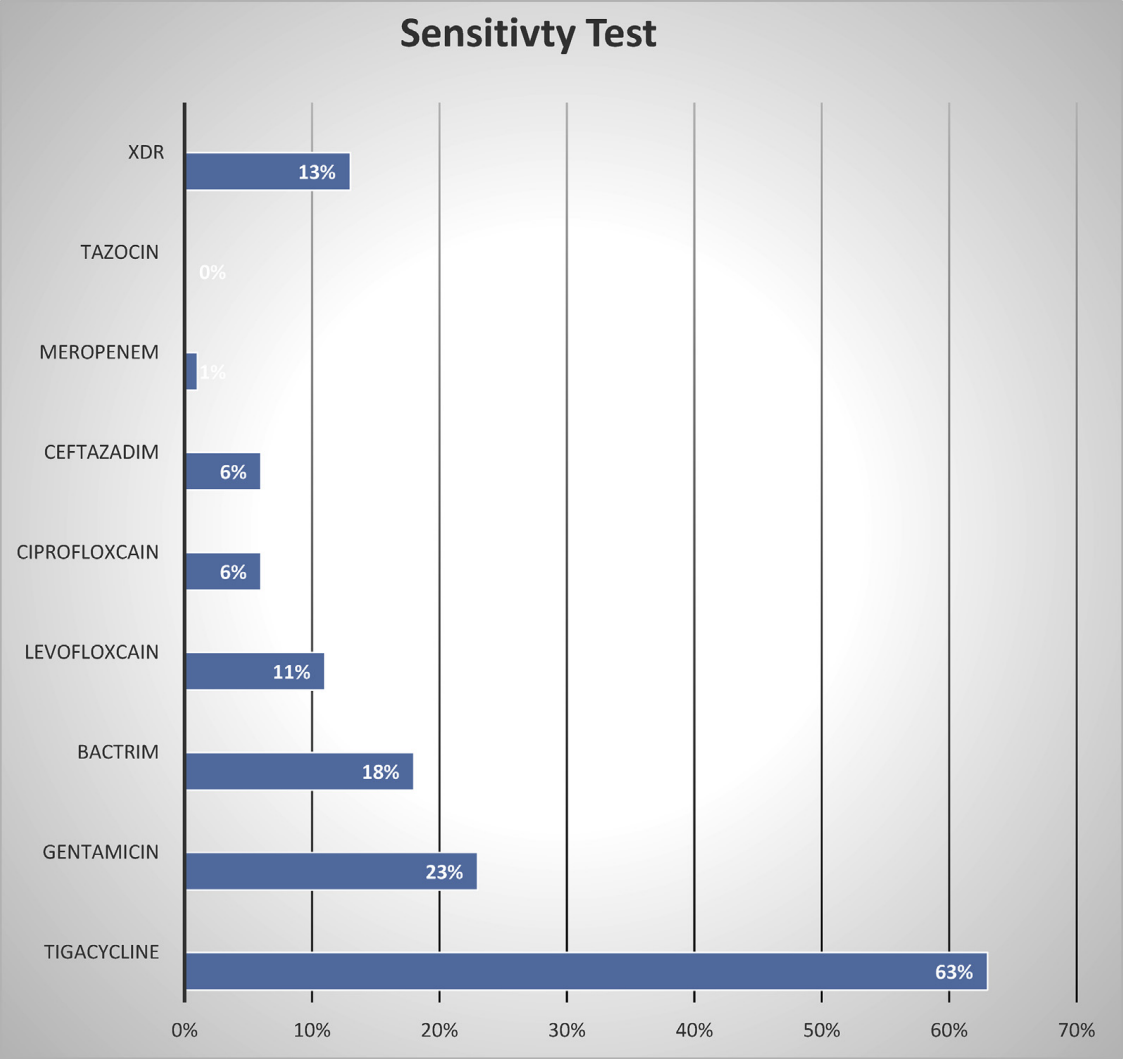
Sensitivity test. XDR, extensively drug-resistant; tazocin (piperacillin/tazobactam); bactrim (trimethoprim/sulfamethoxazole).

The susceptibility tests were done for all positive cultures of *A.bummanii*, which revealed that the sensitivity percentage for each medication as the following: piperacillin/tazobactam (Tazocin) (0%), meropenem (1%), ceftizidime (6%), ciprofloxacin (6%), levofloxacin (11%), tigecycline (63%), gentamicin (23%), and trimethoprim/sulfamethoxazole (Bactrim) (18%) (Figure 3). This indicated that all *A.bummanii* bacterial infections from the study patients were resistant to the available antibiotics at our institution. Tigecycline was the only antibiotic that still remained with a slight sensitivity level above 50%. No test was available for colistin at our institution to determine the sensitivity.

The most commonly used antibiotics either as monotherapy (44%) or in combination (56%) were colsitin followed by meropenem (Table 3). Most of the patients who received combination therapy were on colistin based combination (Table 4).

**Table 3:** Number of antibiotics.

Antibiotics used	No. of patients use these mediation either in combination or mono therapy
Colistin	44
Meropenem	34
Tigecycline	15
Piperacillin/tazobactam	17
Ceftriaxone	6
Imipenem/cilastatin	4
Gentamicin	4
Vancomycin	3
Ceftazidime	3
Ciprofloxacin	2
Levofloxcain	1
Trimethoprim/sulfamethoxazole	1

**Table 4:** Monotherapy vs. Combination Therapy.

Monotherapy (n = 33)	

Colistin	9
Meropenem	9
Piperacillin/tazobactam	6
Tigecycline	3
Ceftriaxone	3
Imipenem/cilastatin	2
Ciprofloxacin	1

Combination Based (n = 40)	

Colistin-based combination	Total = 30
Colistin + Meropenem	10
Colistin + Meropenem + Piperacillin/tazobactam	6
Colistin + Tigecycline	5
Colistin + Meropenem + Tigecycline	5
Colistin + Bactrim	1
Colistin + Gentamicin	1
Colistin + Ciprofloxacin + Piperacillin/tazobactam	1
Colistin + Imipenem/cilastatin + Piperacillin/tazobactam	1
Other combination	Total = 10
Tigecycline + Gentamicin	4
Meropenem + Gentamicin	2
Meropenem + Vancomycin + Piperacillin/tazobactam	1
Meropenem + Ceftriaxone + Levofloxacin	1
Piperacillin/tazobactam + Ceftazidime	1
Piperacillin/tazobactam + Vancomycin + Ceftrixone	1

For patients’ outcomes, the median length of stay at ICU was 32 days. The 14-day survival rate among patients with of *A.bummanii* pneumonia infection was 45%.

## 4 Discussion

*A.baumannii* is a significant gram negative bacteria that causes several types of infections with an increasing rate of resistance. There is only one published study in the Eastern Province. It studied the resistance patterns of *A.baumannii* and its prevalence in the region, which found an increase in the resistance pattern against multiple commonly used antibiotics such as: carbapenems, cephalosporin, and piperacillin/tazobactam [15].

Since there are locally limited studies on this topic in the Eastern Province of Saudi Arabia, this study aims to provide information regarding the risk factors which predispose the patients to this infection and attempt to investigate the most used antibiotic regimen in relationship to survival rate.

At our institution, during the year of 2017, there were 71 ICU patients admitted with pneumonia infection caused by *A.baumannii*. A study that was done in Riyadh showed the most common pathogen associated with HAP and VAP among ICU patients was *A.baumannii* (23.9%) [6]. In our study, we did not compare the incidences of *A.baumannii* with other pathogens that caused pneumonia at our ICUs, but the number of cases were relatively high in this short period of only one year.

The study showed that more than 50% of these patients were mechanically ventilated or use of a catheter during their hospital stay. Other studies have also shown similar risk factors [11, 12, 13]. Most importantly, 84% of our patients received antibiotics 90 days prior to thier infection. All of these are known to be risk factors related to pneumonia caused by *A.baumannii*.

At our institution, testing for a sensitivity panel of *A.baumannii* cultures is available against most antibiotics. However, colistin sensitivity testing is not available at our institution. When a patient has an infection due to extended drug-resistance *A.baumannii*, most clinicians at our institution would select colistin as a main definitive therapy either as monotherapy or combination therapy regardless of unavailability of colistin sensitivity testing. Unfortunately, this study showed that meropenem, which is considered to be one of the main agents for this bacterial infection, was sensitive in only 1% of the *A.baumannii* positive cultures. The most sensitive agent among our patient is tigecycline (63%), which is not routinely recommended to be used for hospital acquired pneumonia. All other antibiotics are under 20% sensitivity.

Most of our patients have combination therapy, 75% of them had a colistin-based combination therapy. Most of the patients with monotherapy received either colistin or meropenem. Meropenem was still continued mostly with another agent even after culture results revealed resistance patterns. In such cases, colistin was the most used agent to be added to the current therapy. A recent randomised controlled study, which was done after collecting our data, concluded that there is no significant difference between colistin monotherapy versus colistin plus meropenem combination as it did not improve the clinical failure in patients with severe *A.baumannii* infections [16].

For our patients, the median length of stay was 32 days. Unsurprisingly, most of our patients died before infection clearance (55%). These results were similar to what was reposted in previous studies [11, 12, 13]. However, the mortality rate cannot be directly correlated to the occurrence of *A.baumannii* pneumonia as these cases are critically ill patients and the cause of death could be attributed to multiple causes.

This study demonstrates poor outcomes associated with pneumonia infection caused by *A.baumannii* among ICU patients. Further improvement should be considered including implementing antimicrobial stewardship and wise use of broad spectrum antibiotics. Also, the newly discovered antibiotics should be considered in such cases. Further studies should be done to assess such implementations as well as use of newer agents and their relationship to patient outcomes.

## Declarations

### Author contribution statement

D. Alsulaiman: Conceived and designed the experiments; Performed the experiments; Analyzed and interpreted the data; Contributed reagents, materials, analysis tools or data; Wrote the paper.

N. Al-Hamed, A. Alziadi, A. Almalaih and M. Alessa: Performed the experiments; Contributed reagents, materials, analysis tools or data; Wrote the paper.

R. Khalil and D. Alshayban: Performed the experiments; Analyzed and interpreted the data; Contributed reagents, materials, analysis tools or data; Wrote the paper.

R. Joseph: Analyzed and interpreted the data; Contributed reagents, materials, analysis tools or data; Wrote the paper.

### Funding statement

This research did not receive any specific grant from funding agencies in the public, commercial, or not-for-profit sectors.

### Competing interest statement

The authors declare no conflict of interest.

### Additional information

No additional information is available for this paper.
